# Streitkulturen – Anmerkungen über Stellungnahmen von Wissenschaftlerinnen und Wissenschaftlern bei kontroversen Debatten

**DOI:** 10.1007/s11616-021-00702-y

**Published:** 2021-12-08

**Authors:** C. Schicha

**Affiliations:** Institut für Theater- und Medienwissenschaft, Universität Erlangen-Nürnberg, Bismarckstr. 1, 91054 Erlangen, Deutschland

Dieser Beitrag widmet sich zunächst einer *Aviso*-Debatte aus dem Jahr 2005. Es ging hierbei u. a. um Stellungnahmen der Wissenschaft zu kontroversen Praxisthemen. Im Anschluss daran skizziere ich Chancen und Risiken von wissenschaftlichen Statements, die auch über Medien publiziert werden können. Es folgt eine Auseinandersetzung mit sogenannten Aufregerthemen und Personen sowie Institutionen, die sich hierzu aus dem wissenschaftlichen Kontext geäußert haben. Abschließend werden Leitlinien und Initiativen vorgestellt, die einen konstruktiven Beitrag zu einer angemessenen Streitkultur bei kontroversen Themen leisten können.

## Rückblick

Expertinnen und Experten aus der Wissenschaft sind gefragt, wenn theoretisches oder praktisches Wissen der jeweiligen Fachdisziplin benötigt wird. Sie artikulieren ihre Kenntnisse und Einschätzungen zu den entsprechenden Themen und liefern damit idealerweise einen konstruktiven Beitrag, um Fachgemeinschaft und Öffentlichkeit korrekt zu informieren. Die normativen Ansprüche an die Vermittlung wissenschaftlicher Wissensbestände sind hoch. Sie sollen relevante Informationen darlegen, die der Aufklärung dienen und formal richtig, rational, aufrichtig und ernsthaft formuliert werden (vgl. Meyer et al. 2001).

Im Februar 2005 wurde in einer Ausgabe der Zeitschrift *Aviso* in mehreren Debattenbeiträgen diskutiert, wie aktuell Wissenschaft sein soll und welche Aufgaben Wissenschaftlerinnen und Wissenschaftler in öffentlichen Diskursen einnehmen sollten. Stephan Alexander Weichert (2005) ging der Frage nach, welchen gesellschaftlichen Gebrauchswert das wissenschaftliche Denken und Arbeiten haben sollte. Er postulierte im Verständnis eines Aufklärungsanspruchs, wissenschaftliche Erkenntnisse schnellstmöglich einem breiteren Publikum zugänglich zu machen, den Anwendungsbezug zu verdeutlichen, mit den Medienakteurinnen und -akteuren den Dialog zu suchen und eine kritische Reflexion der Medienberichterstattung vorzunehmen. Irena Neverla (2005) plädierte am Beispiel der Kriegsberichterstattung dafür, dass speziell die Journalistik kritisch auf aktuelle Entwicklungen reagieren und Leitlinien für journalistisches Handeln vorlegen sollte, um Kriterien für eine bessere journalistische Praxis anzubieten. Horst Pöttker (2005) wies darauf hin, dass zeitliche Aktualität kein Garant für wissenschaftliche Qualität darstelle. Demzufolge sei auch hier die Maxime „Gründlichkeit vor Schnelligkeit“ zu beachten. Gleichwohl soll die Kommunikationswissenschaft, Pöttker zufolge, Handlungs- und Praxisrelevanz anstreben.

Ich habe in der Zeitschrift die These vertreten, dass sich Vertreterinnen und Vertreter von Wissenschaftsdisziplinen zu aktuellen gesellschaftlichen Entwicklungen äußern sollten. Dabei habe ich für einen öffentlichen Austausch in Interviews und Diskussionsrunden ebenso plädiert wie für Statements in Form von Essays oder Kommentaren in Tagezeitungen und Fachorganen (vgl. Schicha 2005).

## Chancen und Risiken wissenschaftlicher Statements in Medien

Daran anknüpfend bin ich 16 Jahre später zu der Erkenntnis gekommen, dass es durchaus riskant sein kann, sich als Wissenschaftler oder Wissenschaftlerin bei kontroversen gesellschaftlichen Debatten über Medien zu Wort zu melden. Widerspruch und Kritik bis hin zum sogenannten Shitstorm können die Konsequenz derartiger Aktivitäten sein.

Aus der Perspektive von Medienmacherinnen und Medienmachern sind Statements von Expertinnen und Experten im O‑Ton angemessen, sofern diese informativ, glaubwürdig, seriös und authentisch arbeiten. Gewünscht sind im Idealfall Informationen aus erster Hand, die durch verständliche Beispiele erklärt werden. Eine einfache Sprache und der Verzicht auf Fremdwörter und Fachtermini sowie der Gebrauch kurzer und unverschachtelter Sätze können dazu beitragen, ein breiteres Verständnis der entsprechenden Ausführungen im Rahmen der Medienberichterstattung zu erreichen.

Provokante Statements können einen Medienbeitrag zwar unterhaltsamer gestalten und Aufmerksamkeit generieren. Spekulationen und Mutmaßungen, die nicht stichhaltig sind, können aber die Seriosität des Experten oder der Expertin in Frage stellen. In Abhängigkeit vom jeweiligen Medium und den Zeitressourcen fehlt es oft am notwendigen Raum, Argumente zu entwickeln, zu erläutern und zu begründen. Daraus resultiert ggf. eine Verkürzung der Aussagen. Zitate können aus dem Zusammenhang gerissen werden und somit nicht intendierte oder missverständliche Botschaften verbreiten. Um dies zu vermeiden, empfiehlt es sich, schriftliche Interviews zu autorisieren. Dennoch können eigene Zitate mit anderen Zitaten vermischt, kommentiert und neu kontextualisiert werden.

Häufige Medienpräsenz von Wissenschaftlerinnen und Wissenschaftlern sowie deren regelmäßige Äußerungen über kontroverse Themen tragen nicht unbedingt dazu bei, die eigene Reputation zu verbessern. Fachkolleginnen und Fachkollegen, die sich in populären Medien äußern, werden abschätzig als sogenannte Medienintellektuelle diskreditiert und erhalten häufig eher Kritik als Zuspruch für ihre Statements (vgl. Schicha 2017). Insgesamt sind Medien also nur bedingt geeignet, eigene Positionen bei kontroversen Themen in angemessener Form argumentativ auszubreiten und zu begründen. Umso wichtiger ist es, derartige Debatten auch in wissenschaftlichen Fachorganen zu führen, die ausreichend Raum und Zeit für Argumente und Gegenargumente sowie Anschlussdiskurse zulassen.

## Aufregerthemen und -akteure

Bei abgesagten kulturellen, politischen und wissenschaftlichen Veranstaltungen und der Löschung von Meinungsbeiträgen ist vielfach von Cancel Culture und Zensur die Rede. Die Unterdrückung unpopulärer Meinungen wird auf der einen Seite als Einschränkung der Meinungs- und Redefreiheit wahrgenommen. Auf der anderen Seite wird bei vermeintlich problematischen Ansichten darauf hingewiesen, dass problematische Einstellungen kein Forum erhalten sollten, um entsprechende Positionen zu verbreiten. Gleichwohl geht es darum, kontroverse Auffassungen öffentlich zu diskutieren (vgl. Buchloh 2003; Ash 2016; Schicha 2019; Schröder 2021).

Wenn Auseinandersetzungen medial aufgegriffen, zugespitzt und personalisiert werden, ist das für die beteiligten Diskutantinnen und Diskutanten bisweilen wenig erfreulich. Dies zeigen u. a. folgende Beispiele:

Der Virologe Christian Drosten (2020) hat durch seine Expertisen während der Corona-Pandemie eine Vielzahl negativer Reaktionen ausgelöst. Er wurde beschimpft und bedroht sowie von Boulevardmedien unter Druck gesetzt.

Gegen die Direktorin des Frankfurter Forschungszentrums Globaler Islam, die Ethnologin Susanne Schröder, hatte es im Vorfeld einer im Jahr 2019 stattfindenden Konferenz an der Universität Frankfurt mit dem Titel „Das islamische Kopftuch – Symbol der Würde oder der Unterdrückung?“ in sozialen Netzwerken Beschimpfungen und Drohungen gegeben. Die Konferenz ist dennoch durchgeführt worden (vgl. Staib 2019).

Dem Politikwissenschaftler Herfried Münkler wurden in dem Blog „Münkler-Watch“ „rassistische, sexistische und militaristische Annahmen und Aussagen in seiner Vorlesung zur politischen Theorie und Ideengeschichte unterstellt“ (Jaeger 2015). Seine Vorlesungen wurden von Protestierenden gestört.

Der Satiriker und Moderator Dieter Nuhr (2020) hat zur Aktion #für das Wissen anlässlich des 100. Geburtstages der Deutschen Forschungsgemeinschaft (DFG) folgende Botschaft formuliert:

Wissen bedeutet nicht, dass man sich zu 100 % sicher ist, sondern dass man über genügend Fakten verfügt, um eine begründete Meinung zu haben. Weil viele Menschen beleidigt sind, wenn Wissenschaftler ihre Meinung ändern: Nein, nein! Das ist normal! Wissenschaft ist gerade, dass sich die Meinung ändert, wenn sich die Faktenlage ändert. Wissenschaft ist nämlich keine Heilslehre, keine Religion, die absolute Wahrheiten verkündet. Und wer ständig ruft „Folgt der Wissenschaft!“, der hat das offensichtlich nicht begriffen. Wissenschaft weiß nicht alles, ist aber die einzige vernünftige Wissensbasis, die wir haben. Deshalb ist sie so wichtig.

Die DFG (2020) hat dieses Statement von Dieter Nuhr nach grundsätzlichen Protesten von der Kampagnenwebsite heruntergenommen. In einem Shitstorm wurde Nuhr u. a. vorgeworfen, dass er den Klimawandel leugne (vgl. Schröder 2021). Später hat die DFG ihre Entscheidung korrigiert und den Beitrag mit folgender Begründung und dem Einverständnis des Künstlers wieder eingestellt:

Die DFG bedauert es ausdrücklich, das Statement von Dieter Nuhr vorschnell von der Internetseite der Online-Aktion #fürdasWissen heruntergenommen zu haben. Herr Nuhr ist eine Person, die mitten in unserer Gesellschaft steht und sich zu Wissenschaft und rationalem Diskurs bekennt. Auch wenn seine Pointiertheit als Satiriker für manchen irritierend sein mag, so ist gerade eine Institution wie die DFG der Freiheit des Denkens auf Basis der Aufklärung verpflichtet. Wir haben den Beitrag daher wiederaufgenommen. Die Diskussion um den Beitrag verdeutlicht exemplarisch die Entwicklungen, die aktuell viele öffentliche Diskussionen um die Wissenschaft kennzeichnen. In verschiedenen Bereichen unserer Gesellschaft hat sich eine Debattenkultur entwickelt, in der oft nicht das sachliche und stärkere Argument zählt, in der weniger zugehört und nachgefragt, sondern immer häufiger vorschnell geurteilt und verurteilt wird. An die Stelle des gemeinsamen Dialogs treten zunehmend polarisierte und polarisierende Auseinandersetzungen. Gerade bei zentralen Fragen wie dem Klimawandel oder der Coronavirus-Pandemie werden damit die wirklich notwendige Diskussion um wissenschaftliche Themen und der konstruktive Austausch zwischen Wissenschaft und Gesellschaft behindert. Wissenschaftlerinnen und Wissenschaftler, die ihre Erkenntnisse öffentlich machen und politische Handlungsoptionen beschreiben, sind immer häufiger Ziel unsachlicher Attacken und persönlicher Diffamierungen. Dies gilt auch für gesellschaftliche Bewegungen, die für die Wissenschaft eintreten und öffentlich dazu aufrufen, wissenschaftliche Erkenntnisse stärker zur Basis von Entscheidungen und Handlungen zu machen. Diese Entwicklungen sind der Gesellschaft nicht zuträglich und umso bedenklicher, als die Wissenschaft bei der Bewältigung aktueller Herausforderungen eine zentrale Rolle spielt, mit der sie derzeit in der Gesellschaft stark wahrgenommen und geschätzt wird. Dabei ist sie ihrerseits auf eine kritische, offene und konstruktive Kommunikationskultur angewiesen.

Die DFG möchte diese Beobachtungen zum Anlass nehmen, eine intensive Auseinandersetzung mit der aktuellen Debattenkultur rund um die Wissenschaft anzustoßen. Die DFG steht für Meinungsvielfalt und Meinungsfreiheit sowie für eine differenzierte Diskussionskultur. Hierfür wird sie sich auch in Zukunft weiter mit aller Kraft einsetzen – gemeinsam mit anderen Akteuren aus Wissenschaft, Medien, Politik und anderen Bereichen der Gesellschaft im In- und Ausland.

Die Korrektur der Löschung des Beitrages von Nuhr und dieses Statement der DFG verfolgen meiner Auffassung nach einen konstruktiven Ansatz, der kontroverse Debatten zulässt und ein Plädoyer für die Meinungsvielfalt liefert. Es wird zu Recht eine offene und argumentative Diskurskultur postuliert, wie sie Habermas und andere als regulative Idee gefordert haben.

## Theorie und Praxis

Bei Blick auf die regulative Idee einer vernunftbasierten Kommunikation in Diskursen gilt nach Habermas:

Die Logik der Argumentation erfordert einen begrifflichen Rahmen, der dem Phänomen des eigentümlich zwanglosen Zwangs des besseren Arguments Rechnung zu tragen erlaubt. (Habermas 1985, S. 52–53)

Eine angemessene gesellschaftliche Kommunikation in der medial vermittelten Öffentlichkeit setzt weiterhin voraus, dass soziale Beziehungen wechselseitiger Anerkennung auf der Basis der formulierten Geltungsansprüche auf Wahrheit, Wahrhaftigkeit und Richtigkeit im Diskurs geprüft und diskutiert werden.

Das aber gelingt nur, wenn man die Argumente wägt und Stellung bezieht, um gegebenenfalls im weiteren Diskurs etwaige Unklarheiten auszuräumen. (Brosda 2019, S. 31)

Dabei sollten wissenschaftliche Erkenntnisse, die sich aufgrund neuer Zahlen, Daten und Fakten ändern können, als vernünftige Grundlage akzeptiert werden.

In der Praxis laufen öffentliche Diskurse hingegen vielfach weniger nach dem Prinzip der maximalen Verständigungsorientierung auf der Basis rationaler Argumente ab. Faktisch geht es neben Machtaspekten auch um Provokation und Polemik, die Verwendung von Stereotypen sowie Ausgrenzung bis hin zum Hass (vgl. Brodnig 2016; Künast 2017). Dabei spielen die sogenannten sozialen Netzwerke eine zentrale Rolle.

Insbesondere die Netzwerk- und Video-Plattformen schaffen Kommunikationsräume, in denen der verständigungsorientierte Austausch von Argumenten nicht im Vordergrund steht, sondern wo sich Menschen wechselseitig in ihrer vorgefassten Meinung bestätigen. Diese Echokammern, in denen nur noch zu Gehör kommt, was dem Gruppenkonsens entspricht, sind vor allem dann problematisch, wenn sie um externe, intolerante und undemokratische Haltungen herum entstehen und die Meinungsäußerungen die Grenzen des zulässigen Streitens gar überschreiten. (Schmidt 2017, S. 86)

*Die grosse Gereiztheit* ist demzufolge der passende Titel eines Buches von Bernhard Pörksen. Es trägt den Untertitel *Wege aus der kollektiven Erregung*. Der Autor diagnostiziert bei öffentlichen Debatten einen „kommentierenden Sofortismus“ (Pörksen 2018, S. 52). Unmittelbar und schnell werden Sachverhalte häufig ohne Prüfung kommentiert und bewertet. Die sogenannten vernetzten Vielen des digitalen Zeitalters haben Pörksen zufolge als fünfte Gewalt eine enorme publizistische Macht erreicht. Im Verständnis von Protestgemeinschaften agieren sie als Medienkritikerinnen und Medienkritiker u. a. auf Blogs, Wikis und sozialen Netzwerken. Die fünfte Gewalt, ergänzt Schulz (2016, S. 57),

[…] tritt eben keineswegs nur in Gestalt einer Kontroll- und Gegenmacht auf, die berechtigte Ansprüche vorträgt und zu Unrecht marginalisierten Positionen eine verdiente Aufmerksamkeit verschafft. Hinter ihr verbergen sich oft auch Penetranz, Populismus, Extremismus, Dilettantismus, Vorurteile, Verschwörungstheorien, Desinformationen, Propaganda und Mobbing.

In dem von Stephan Russ-Mohl (2020) herausgegebenen Sammelband *Streitlust und Streitkunst* mit dem Untertitel *Diskurs als Essenz der Demokratie* wird im Text auf dem Buchrücken konstatiert, dass sich die öffentliche Kommunikation angesichts von Corona-Pandemie, Migrationskrise und Klimakatastrophe zunehmend polarisiere (vgl. hierzu auch Brosda 2020). Weiter heißt es dort:

Sie wird schriller und der Umgangston rauer, ja oft unerträglich. Auf der Strecke bleibt nicht die Streitlust, wohl aber die Streitkunst, die in der Tradition der Aufklärung nach tragfähigen politischen Kompromissen in unseren Demokratien sucht.

Es wird die These vertreten, dass der „übergreifende öffentlich-demokratische Diskurs gefährdet“ sei. Ihm zufolge ist der „öffentliche Wettbewerb auf dem Marktplatz der Ideen ins Stocken geraten“. Der Herausgeber verfolgt die ambitionierte Absicht, mit seinen Schriften einen Beitrag „zur Rettung des öffentlichen Diskurses“ zu leisten.

Der Journalist Milosz Matuschek und der Philosoph Gunnar Kaiser (2020) haben in einer Petition einen Appell für freie Debattenräume initiiert. Sie gehen davon aus, dass in Deutschland die Meinungsfreiheit u. a. aufgrund der Ausladung von Kabarettistinnen und Kabarettisten, gestörten Seminaren und Vorlesungen sowie verbotenen Demonstrationen gefährdet sei. Dort heißt es u. a.:

Absagen, löschen, zensieren: seit einigen Jahren macht sich ein Ungeist breit, der das freie Denken und Sprechen in den Würgegriff nimmt und die Grundlage des freien Austauschs von Ideen und Argumenten untergräbt. Der Meinungskorridor wird verengt, Informationsinseln versinken, Personen des öffentlichen und kulturellen Lebens werden stummgeschaltet und stigmatisiert. […] Die gezielte Verunglimpfung von Intellektuellen, Künstlern, Autoren und jedem, der von der aktuell herrschenden öffentlichen Meinung abweicht, ist eine inakzeptable Anmaßung. Freie Rede und Informationsgewinnung sowie freie wissenschaftliche oder künstlerische Betätigung sind Rechte und nicht Privilegien, die von dominierenden Gesinnungsgemeinschaften an Gesinnungsgleiche verliehen und missliebigen Personen entzogen werden können. Es ist dabei unerheblich, auf welcher politischen Seite die Gruppierung steht, ob sie religiös, weltanschaulich oder moralisch motiviert ist – ein Angriff auf die Demokratie bleibt ein Angriff auf die Demokratie.

Unterzeichnet haben den Aufruf u. a. der Tübinger Oberbürgermeister Boris Palmer, die Schriftstellerin Monika Makron, der Satiriker Dieter Nuhr, der Historiker Götz Aly, der Medienwissenschaftler Norbert Bolz und die Journalisten Harald Martenstein und Günter Wallraff (vgl. Krischke 2020).

Insofern gibt es dort und auch in weiteren Medienformaten zahlreiche Debattenräume, die den Anspruch erheben, kontroverse Diskurse auf der Basis gut begründeter Argumente zu führen.

## Streitkultur in den Medien und der Wissenschaft

Faktisch hat sich das Feld der Diskutantinnen und Diskutanten in der öffentlichen Kommunikation erweitert. Bürgerinnen und Bürger können als sogenannte Prosumer Inhalte nicht nur konsumieren, sondern unmittelbar über diverse Onlinekanäle kommentieren. Es ist einfach und kostengünstig, den eigenen Standpunkt virtuell zu artikulieren und Anschlussdiskurse zu generieren. Somit besteht die Möglichkeit, einen Betrag zur Meinungs- und Willensbildung zu leisten. Der klassische Journalismus hat sein Gatekeepermonopol verloren, und sogenannte Influencer, denen auch vorgeworfen wird, antiaufklärerisch zu agieren und ihre Follower zu manipulieren, beteiligen sich an öffentlichen Debatten (vgl. Nymoen und Schmitt 2021). Der Youtuber Rezo etwa veröffentlichte kurz vor der Europawahl im Mai 2019 ein 55-minütiges Video mit dem Titel „Die Zerstörung der CDU“, das rund 17 Mio. Klicks erreichte (vgl. Brosda 2019). Weitere Videos, in denen er einzelne Politiker und Politiker aufgrund ihrer Nähe zur Industrie kritisierte, folgten. Rezo kritisierte im Mai 2020 zudem klassische Medien wie die *Welt* und die *Frankfurter Allgemeine Zeitung* im Video „Die Zerstörung der Presse“ aufgrund falscher Behauptungen und journalistischer Fehler (vgl. Reuter 2020).

In der Ausgabe der Wochenzeitschrift *Die Zeit* vom 5. September 2019 wurde das Ressort „Streit“ eingeführt. Seither gibt es im Blatt eine „Anklage“ und eine „Verteidigung“. Gewünscht ist eine kontroverse, aber respektvolle Auseinandersetzung über gesellschaftlich relevante Themen wie Migration, Feminismus und Religion, die sachliche Argumente umfasst und unterschiedliche Positionen deutlich macht.

Auch die DGPuK setzt sich zu Recht mit aktuellen Medienentwicklungen auseinander und reflektiert dabei normative Aspekte. Die Fachgruppe Kommunikations- und Medienethik etwa hat sich auf ihrer Jahrestagung 2021 mit der „Ethik der Streitkulturen“ auseinandergesetzt und wird sich auf der Jahrestagung im Februar 2022 mit „ethischen Anforderungen an die Kommunikation zwischen Wissenschaft und Gesellschaft“ beschäftigen.

## Vorschläge zur Verbesserung der Streitkultur

Die Austragung von politischen Meinungsunterschieden gehört wesentlich zum demokratischen Prinzip. (Frick 2017, S. 89)

Es ist grundsätzlich begrüßenswert, kontroverse Debatten öffentlich zu führen. Dabei ist es notwendig, „Zusammenhänge zu erklären“ (Künast 2017, S. 177) und unfaire Diskussionsstile zu problematisieren. Carsten Brosda (2019, S. 15) plädiert dafür, „radikal verständigungsbereit“ und „leidenschaftlich vernünftig“ zu sein. Er setzt auf folgende Kriterien, um angemessene Diskurse zu bewerkstelligen:

- Kritische Debatte: Probleme benennen, Fehler ansprechen, zuhören, argumentieren
- Klarheit: Keine Verwendung strategischer oder verschleiernder Begriffe oder Metaphern
- Konsistenz: Sprechen und Handeln in Einklang bringen
- Kontinuität: Grundbotschaften entwickeln und ausdauernd wiederholen
- Kohärenz: grundlegende Werteframes entwickeln und kommunizieren
- Kooperation: öffentlicher und kommunikativer Schulterschluss mit Partnern, die Werte und Ziele teilen
- Kampagne: Aktions- und kampagnenorientierte Kommunikationsformen entwickeln (vgl. Brosda 2019, S. 161–162)

Die Philosophin Romy Jaster und der Philosoph David Lanius (2017) haben die Idee eines Forums für Streitkultur entwickelt, das zehn Regeln für eine gute Debatte umfasst, um Eskalationen zu vermeiden. Dazu gehören der Versuch, den Gesprächspartner oder die Gesprächspartnerin richtig zu verstehen, beim Thema zu bleiben, Fragen zu stellen, Gemeinsamkeiten zu finden, Belehrungen zu unterlassen, den eigenen Standort zu begründen, die Position des Gegenübers wohlwollend zu interpretieren, aber sachliche Kritik zu üben, ruhig zu bleiben und zu deeskalieren sowie die eigene Position zu wechseln, um den Gesprächspartner oder die Gesprächspartnerin besser verstehen zu können.

Aus einer journalistischen Perspektive hat Jeff Jarvis (vgl. Ellers 2020) weitere Empfehlungen vorgestellt, um Polarisierungen in öffentlichen Debatten im digitalen Zeitalter zu verringern. Dazu gehören bessere Möglichkeiten, einander zuzuhören, vertrauenswürdige und verlässliche Quellen zu stärken, eine stärkere Vernetzung zwischen Journalismus und Wissenschaft zu fördern, sowie „Fake-News“, Verschwörungserzählungen und alternative Fakten zu widerlegen, um produktive Formen der Auseinandersetzung zu fördern (vgl. dazu auch Schicha et al. 2021).

## Fazit und Ausblick

Wir benötigen einen Diskurs, einen niveauvollen, also fair und mit Sachargumenten geführten Streit um gemeinsame Belange, Werte und Normen des Zusammenlebens. (Stapf et al. 2017, S. 11)

Mediendiskurse sind im Wandel begriffen und unterliegen permanenten Veränderungen. Es ist zu prüfen, inwiefern derartige Diskurse den normativen Anforderungen eines respektvollen Miteinanders genügen, an welchen Stellen Überschreitungen und Grenzverletzungen der sogenannten guten Sitten zu beobachten sind, welche Akteure sich auf welchen Foren in welcher Weise öffentlich artikulieren und welche Folgen dies aus einer medienethischen Perspektive haben kann. Grundsätzlich herrscht in vielen Fällen ein Spannungsfeld zwischen dem Recht auf freie Meinungsäußerung und den damit einhergehenden negativen Konsequenzen für die Betroffenen vor, zumal vielfach auch mit Klischees, Vorurteilen, Stereotypen und unzulässigen Verallgemeinerungen im Rahmen von Debatten gearbeitet wird. Polarisierende Diskurse, die Desinformationen verbreiten, können hierbei die Folge sein. Provokationen, Polemiken und Tabubrüche prägen neben Beschimpfungen auch öffentliche Debatten. Es stellt sich die Frage, wie eine vernünftige, verständigungsorientierte, ernsthafte, respektvolle, tolerante und demokratische Form der Kommunikation unter veränderten digitalen Bedingungen aus einer normativen Perspektive bewerkstelligt werden sollte, um Konflikte konstruktiv zu bewältigen. Die Aufgabe besteht darin, konstruktiv mit der wachsenden Vielfalt von widersprüchlichen Meinungen umzugehen. Wechselseitige kommunikative Bezüge sind hierbei unverzichtbar, um eine produktive Streitkultur zu bewerkstelligen.

Grundsätzlich sollten kontroverse Debatten auf allen öffentlichen Ebenen durch schriftliche und verbale Statements aller am Diskurs beteiligten Diskutantinnen und Diskutanten sowohl über die Medien als auch innerhalb der Wissenschaftsformate auf der Basis der Leitlinie von Habermas (1985) so ausgetragen werden, dass es im Idealfall keinen Zwang außer dem Zwang des besseren Argumentes gibt.

Die Qualität einer konstruktiven Streit- und Debattenkultur hängt schließlich zentral davon ab, dass sich eine Vielzahl von qualifizierten Expertinnen und Experten äußern. Es geht schließlich darum, nicht nur Diskursvermittlung zu betreiben, sondern eine eigenständige Rolle als Diskursteilnehmer oder Diskursteilnehmerin zu übernehmen, um damit eine Vielfalt qualifizierter Beiträge zu initiieren, die für einen demokratischen Austausch von Argumenten zentral sind. Insofern sollte die Maxime „Mehr Diskurs wagen“ auch für die Wissenschaft gelten.

Insofern möchte ich dafür plädieren, dass auch die *Publizistik* weiterhin kontroverse Themen wie die sogenannte Genderdebatte aufgreift und unterschiedlichen Meinungen und Positionen auf der Basis begründeter Argumente einen angemessenen Raum bietet. Der Aufsatz von Rudolf Stöber (2020) über „Genderstern und Binnen‑I. Zu falscher Symbolpolitik in Zeiten eines zunehmenden Illiberalismus“ in der *Publizistik* etwa hat sowohl die Kritik von Thomas Hanitzsch (2021) als auch einen offenen Brief zahlreicher Wissenschaftlerinnen und Wissenschaftler an den Vorstand der Deutschen Gesellschaft für Publizistik und Kommunikationswissenschaft (o. V. 2021) ausgelöst, in dem ein „Versagen wissenschaftlicher Qualitätssicherung und redaktioneller Verantwortung“ konstatiert worden ist. Daraufhin haben die Herausgeberinnen und Herausgeber der *Publizistik* (2021) geantwortet und ihre Argumente für die Aufnahme des Stöber-Textes dargelegt. Derartige Kontroversen offen auszutragen ist aus meiner Sicht grundsätzlich positiv zu bewerten. Somit wird das breite Spektrum unterschiedlicher Haltungen und Positionen von Wissenschaftlerinnen und Wissenschaftlern über relevante Themenfelder dokumentiert. Zudem werden Anschlussdebatten generiert, die weitere Diskursräume öffnen.
